# Numerical Magnitude Affects Accuracy but Not Precision of Temporal Judgments

**DOI:** 10.3389/fnhum.2020.629702

**Published:** 2021-01-15

**Authors:** Anuj Shukla, Raju S. Bapi

**Affiliations:** Cognitive Science Lab, Kohli Research Centre on Intelligent Systems, International Institute of Information Technology, Hyderabad, India

**Keywords:** numerical magnitude, temporal perception, Weber ratio, temporal experience, temporal bias

## Abstract

A Theory of Magnitude (ATOM) suggests that space, time, and quantities are processed through a generalized magnitude system. ATOM posits that task-irrelevant magnitudes interfere with the processing of task-relevant magnitudes as all the magnitudes are processed by a common system. Many behavioral and neuroimaging studies have found support in favor of a common magnitude processing system. However, it is largely unknown whether such cross-domain monotonic mapping arises from a change in the accuracy of the magnitude judgments or results from changes in precision of the processing of magnitude. Therefore, in the present study, we examined whether large numerical magnitude affects temporal accuracy or temporal precision, or both. In other words, whether numerical magnitudes change our temporal experience or simply bias duration judgments. The temporal discrimination (between comparison and standard duration) paradigm was used to present numerical magnitudes (“1,” “5,” and “9”) across varied durations. We estimated temporal accuracy (PSE) and precision (Weber ratio) for each numerical magnitude. The results revealed that temporal accuracy (PSE) for large (9) numerical magnitude was significantly lower than that of small (1) and identical (5) magnitudes. This implies that the temporal duration was overestimated for large (9) numerical magnitude compared to small (1) and identical (5) numerical magnitude, in line with ATOM’s prediction. However, no influence of numerical magnitude was observed on temporal precision (Weber ratio). The findings of the present study suggest that task-irrelevant numerical magnitude selectively affects the accuracy of processing of duration but not duration discrimination itself. Further, we argue that numerical magnitude may not directly affect temporal processing but could influence *via* attentional mechanisms.

## Introduction

Processing of space, time, and the number has been an integral part of human cognition. Our day-to-day actions and behaviors are very much contingent on the processing of these magnitude dimensions. For example, from complex behavior such as driving a car to a simple task like grabbing five sheets of paper from the table, both require precision in processing space, time, and number to execute our actions effectively. Often, we feel that the presence of one magnitude dimension influences the processing/judgment of other dimensions.

In earlier studies, time, space, number, and other magnitude-related processes in the mind/brain have been studied extensively and independently. Based on the findings from the studies in these magnitude domains, a popular theoretical framework was proposed by Walsh (2003). This framework is called “A Theory of Magnitude (ATOM).” ATOM proposes a generalized magnitude system for all kinds of magnitude-related processing in the brain. Specifically, ATOM states that a shared common mechanism supports time, space, and number processing. One of ATOM’s predictions is that of a monotonic mapping across different magnitude systems, i.e., the lesser magnitude in one domain (say, smaller duration) will be associated with the lesser magnitude in another domain (say, a smaller number, for example), and the same goes for larger magnitudes as well. ATOM theory extrapolates from these correlated monotonic mappings that different magnitude dimensions influence one another during the processing stage. In the past two decades, many behavioral studies have gathered evidence in favor of a generalized magnitude system and argued for the presence of a common magnitude system (Xuan et al., 2007; Srinivasan and Carey, 2010; Cai and Connell, 2015; Schwiedrzik et al., 2016; Yamamoto et al., 2016). On the contrary, more recent studies have provided evidence for independent processing of these magnitude domains and argued against a generalized magnitude system (Dormal et al., 2006, 2008; Agrillo et al., 2010; Young and Cordes, 2013; Hamamouche et al., 2018). Apart from the behavioral studies, a handful of neuroimaging studies have also supported the idea of common magnitude processing and reported that cross-domain magnitude interaction takes place in the prefrontal and parietal cortices in the brain (Hubbard et al., 2005; Bueti and Walsh, 2009; Hayashi et al., 2013a; Skagerlund et al., 2016). It has been argued that such cross-domain magnitude interaction may also result from automatic analogical processing when magnitudes from different dimensions are processed together. Such analogical processing is also represented in the frontal and parietal brain regions (for related discussion see, Bunge et al., 2005; Speed, 2010; Vicario and Martino, 2010).

In addition to neuroimaging studies, growing evidence from the clinical population, e.g., Developmental Dyscalculia (DD) and children with chromosome 22q11.2 syndrome also provide support to a generalized magnitude system (Simon, 2008; Hurks and Loosbroek, 2012; Vicario et al., 2012, 2013). Specifically, Simon (2008) suggested that children with DD and 22q11.2 syndrome face difficulty processing magnitudes like space, time, and number compared to typically developing children because of possible dysfunction in the neural substrates responsible for processing spatiotemporal information.

Behavioral studies investigating the influence of task-irrelevant numerical magnitude on temporal processing have demonstrated that the duration for a large numerical magnitude tends to be overestimated, and the duration for a small numerical magnitude is underestimated. Participants were presented a target number (“1,” “5” and “9”) with varied durations against a fixed reference number (“5”) associated with a fixed reference duration. Participants were required to make a forced judgment as to whether the target number lasted longer or shorter compared to the reference. In line with ATOM’s prediction, participants overestimated the duration of a large number and underestimated the duration of a small number (Oliveri et al., 2008).

Furthermore, to investigate whether the number influences duration judgment at the perceptual level, Chang et al. (2011) used a temporal reproduction task. The findings suggest that the large numerical magnitude led to the reproduction of longer durations than the small numerical magnitude when presented at the encoding stage. On the contrary, participants reproduced a shorter duration for large numerical magnitude than small numerical magnitudes when presented at the reproduction stage. The authors interpreted the modulation in the perceived duration due to the influence of a common numerical magnitude representation at the encoding stage, in line with ATOM. Further, as an alternate explanation, the authors also speculated that such stage-dependent influence of numerical magnitude on temporal processing may reflect the differential effect of numerical magnitude on the speed of the internal clock at the encoding and reproduction stages. However, a recent study rejects the internal clock account that assumes that large magnitudes speed-up an internal clock at the encoding stage (Cai and Wang, 2014).

Previous studies investigating the influence of number on time have also documented a pronounced contextual-dependency (Lu et al., 2009; Vicario, 2011) and gender-related differences (Hayashi et al., 2013b) in number-time interaction. Additionally, few studies investigated the role of attention in size-magnitude and time interaction by using a dual-task paradigm and demonstrated a positive relationship between duration reproduction and the magnitude size (see Rammsayer and Verner, 2014, 2015).

In contrast, more recent studies have provided substantial evidence against the proposal of a common magnitude system and argued for *domain-specific processing*. In a recent study using a temporal bisection task, participants were tested on numerosity and temporal judgments under dual-task conditions. They remembered letters while making temporal or numerical judgments. Authors hypothesized that if a common magnitude processing were operating, both number and time would exhibit similar biases even under the cognitive load condition. However, the result shows that cognitive load leads to differential biases across the two magnitude domains. More specifically, in the cognitive load condition, participants underestimated numerosity in the numerosity task, whereas they overestimated duration in the temporal judgment task (Hamamouche et al., 2018). Similar results have also been seen when participants were asked to make judgments about time and numerosity under the influence of emotion (Young and Cordes, 2013). Such differential effects on temporal perception across numerical magnitudes show that the processing of numbers and time may not be mediated by a common magnitude processing system. Taken together these inconsistent findings cast doubt on the existence of a generalized magnitude system for space, time, and number.

It is important to note that studies arguing in favor of the common magnitude system have shown overestimation of duration for a large magnitude and underestimation of duration for a small magnitude with respect to each other. For example, the relative overestimation of time for the large magnitude has always been typically reported in the context of a small numerical magnitude. However, it may be possible that such relative temporal processing difference can be observed due to differential cognitive demands involved in the processing of small and large numerical magnitudes and may not necessarily be modulated by a common magnitude system. Thus, the fundamental question that needs to be asked is whether numerical magnitudes affect duration genuinely. Suppose a common magnitude system processes time and number dimensions. In that case, the small numerical magnitude should elicit more “short” responses, and the large numerical magnitude should generate more “long” responses, which ultimately leads to underestimation and overestimation of duration, respectively. It would be particularly interesting if a large numerical magnitude elicits more “long” responses for the given objective duration than that of a small numerical magnitude. This would suggest that numerical magnitude not only biases our temporal judgments but also affects the overall experiences of duration itself. Consequently, it makes sense how the duration associated with a large numerical magnitude is perceived to be longer than that associated with a small numerical magnitude. So far it is not clear whether numerical magnitudes change our temporal experience or simply bias our duration judgments. Therefore, in the present article, we investigate whether the task-irrelevant numerical magnitude interacts with temporal processing by influencing temporal accuracy or temporal precision, or both.

To examine the above objective, we experimented using a temporal discrimination task wherein a task-irrelevant numerical magnitude was presented for a varied duration. Participants were asked to judge the duration of the numerical magnitude. We hypothesize that if numerical and temporal information are processed through a common magnitude system, the large numerical magnitude would elicit more “long” responses than the small numerical magnitude for a given duration, thus resulting in the overestimation of duration for large numerical magnitude. Similarly, the small numerical magnitude would elicit more “short” responses than that of large numerical magnitude for a particular duration and in turn, lead to the underestimation of duration for small numerical magnitudes.

## Methodology

### Apparatus

The stimuli were presented and controlled using E-Prime Standard-2.0 (Schneider et al., 2002) on a 17’’ CRT monitor (1,024 × 768 resolution) running at a frame rate of 100 Hz.

### Participants

Twenty-seven participants (15 males; age range 20–27 years) were recruited from the International Institute of Information Technology, Hyderabad, India. All the participants had normal or corrected-to-normal vision. The study was approved by the Institute Review Board (IRB), International Institute of Information Technology, Hyderabad, India. Participants gave written informed consent before the experiment. They received remuneration against their participation.

### Stimulus

**Figure 1:**
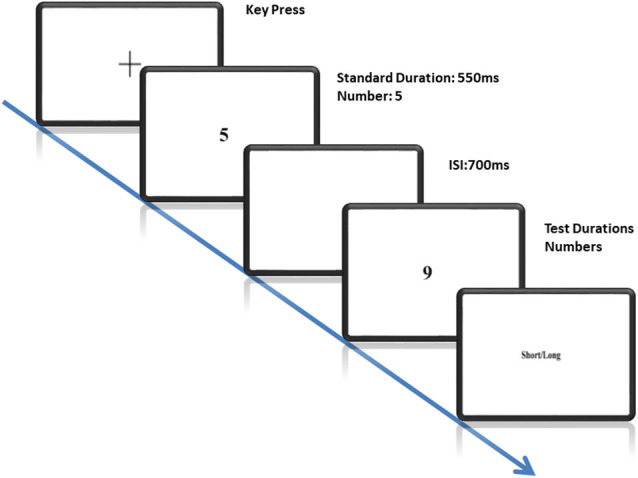
Illustration of the Task: each trial starts with the fixation cross followed by a standard stimulus with a fixed duration and subsequently a comparison stimulus with variable durations and numbers. The standard and the comparison stimuli were separated by an interstimulus interval of 700 ms. Participants were required to compare whether the comparison stimulus lasted longer as compared to the standard stimulus.

The experiment began with a fixation cross presented at the center of the monitor. Participants were asked to press the spacebar to start a new trial. Black stimuli (numerals) were presented on a white background. The trial starts with a fixation cross followed by a standard stimulus with fixed duration followed by a comparison stimulus presented with varying durations. An Inter-Stimulus Interval (ISI) of 700 ms was used to separate the standard and the comparison stimuli (see Figure 1). Participants were informed that they would be shown a standard duration with the number “5” followed by comparison durations with numbers “1,” “5” or “9.” They were required to judge whether the comparison stimuli lasted longer or shorter than the standard stimulus in every trial. They were asked to make their duration judgments independent of the presented magnitudes. Participants executed their response by pressing a dedicated key (“L” for long and “S” for short) on the keyboard for “long” and “short” responses. The response keys were counterbalanced across participants.

### Design and Procedure

In the current study, we used three numerals: “5” being the reference magnitude, “1” being small, and “9” being large comparison magnitudes. In “identical” trials, the reference and comparison were of the same magnitude. These numbers were displayed with a 2° visual angle. We took seven objective durations from 250 to 850 ms with steps of 100 ms and a fixed standard duration of 550 ms. Participants were taken to a dimly-lit experimental room. They were asked to sit comfortably. The distance between the participant and the computer monitor was 57 cm. Instructions were given in both verbal and written format. All participants received 10 practice trials before starting the main experiment. The durations used in the practice trials were different from the durations used in the main experiment. Each duration was repeated seven times for each numerical magnitude constituting a total of 147 trials per participant.

## Results

Out of 27 participants, data from three participants had to be removed from the final analysis as their data could not be fit to the psychometric function.

### Does Numerical Magnitude Actually Elicit More “Long”/“Short” Responses?

Previous studies using temporal reproduction tasks have shown that participants reproduced longer durations in the presence of large magnitude and shorter duration for small magnitude. Such results have allowed researchers to believe in a cross-domain monotonic relation between the number and duration dimension. To test the cross-domain monotonic relation between numerical magnitude and durations, we pooled the durations into *Short Duration*, *Same Duration*, and *Long Duration*. The durations below the standard (550 ms) were binned as “short duration” and those above the standard (550 ms) were binned as “long duration.” When the standard (550 ms) duration was used as standard as well as comparison duration we call it the “same duration.” The *average proportion of long responses* [hereafter denoted as *p(long)*] were computed for each numerical magnitude across the three durations and were analyzed using a robust analysis, the rank-based ANOVA-type statistic (Noguchi et al., 2012). To evaluate whether large numerical magnitude generated more long responses and small numerical magnitude more short responses, a 3 (Magnitude: Small, Identical, and Large) × 3 (Duration: Short, Same, and Long) within-subject repeated measures ANOVA-type analysis was used. Given the previous findings in similar settings, one can expect that if the number and time have a monotonic relation, combining short duration with a small number would elicit more “short” responses for the given duration. Similarly, combining long durations with a large number would elicit more “long” responses.

The 3 × 3 repeated measure ANOVA-type statistic revealed a main effect of duration on the proportion of long responses (*F*_(1.88,∞)_ = 320.57, *p* < 0.05). This suggests that the *p*(*long*) responses systematically increased with increased duration. The *post hoc* analysis suggested that Short (0.118 ± 0.10; mean ± SD), Same (0.516 ± 0.25), and Long (0.851 ± 0.12) durations were statistically different from one another (*p* < 0.05), indicating that short durations were judged shorter and long durations were judged longer. Further, the results also suggested a main effect of magnitude (*F*_(1.99,∞)_ = 12.94, *p* < 0.05). The *post hoc* analysis indicated that the mean *p(long)* responses for small magnitude (0.479 ± 0.34) and large magnitude (0.547 ± 0.34) were found to be significant (*p* < 0.005). Similarly, the *p(long)* responses for identical magnitude (0.459 ± 0.35) and large magnitude (0.547 ± 0.34) were also significant (*p* < 0.05). However, the *p(long)* responses for the small (0.479 ± 0.34) and identical magnitude (0.459 ± 0.35) were not found to be statistically significant (*p* > 0.05). This indicates that large numerical magnitude elicited more long responses compared to identical and small numerical magnitudes. However, we did not observe Magnitude × Duration interactions (*F*_(2.38,∞)_ = 0.071, *p* > 0.05). This insignificant interaction suggests that the *p(long)* responses for the magnitude were not different across durations. In other words, large numerical magnitude did not elicit more “long” responses than that of small or identical magnitudes on the given durations.

### Does Numerical Magnitude Affect Temporal Perception?

**Figure 2:**
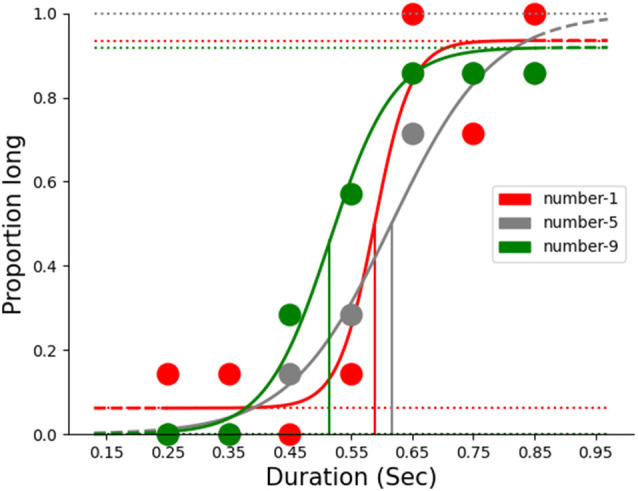
Psychometric fit for the results of a representative subject.

Further, we plotted the average proportion of long responses, *p(long)* across probe durations (250–850 ms) for each numerical magnitude and fitted a logistic function using *psignifit-4*, a MATLAB-based toolbox to estimate the point of subjective equality (PSE). PSE is the point on the psychometric fit where the frequencies of long and short responses are found to be the same (i.e., 50%). PSE is also considered as the *accuracy of temporal judgments*. A leftward shift of the psychometric curve indicates overestimation of duration and a rightward shift of the curve an underestimation of duration (see Figure 2). We estimated the PSE values for each numerical magnitude across the participants using the logistic function and the model fit was assessed for all the numerical magnitudes ($R_{\left(\text{small}\right)}^{2}$ = 0.92 ± 0.07; $R_{\left(\text{identical}\right)}^{2}$ = 0.93 ± 0.08; $R_{\left(\text{large}\right)}^{2}$ = 0.93 ± 0.05).

To test whether the numerical magnitudes affected temporal perception, we submitted the estimated PSE values for each magnitude to a one-way repeated measures ANOVA. The analysis yielded a significant main effect, thereby indicating that the PSE values differed significantly across the numerical magnitudes (*F*_(2,46)_ = 10.23, *p* < 0.001, η^2^ = 0.30). Further, the *post* *hoc* test (*holm’s*) suggested that duration judgments associated with large numerical magnitude (523.54 ± 65.8 ms) were significantly overestimated than those with small (554.88 ± 79.8 ms) and identical (582.66 ± 84.8 ms) magnitudes (*p* < 0.05). This suggests that temporal perception may be affected by the numerical magnitude that was presented conjoin.

In the time perception literature, temporal overestimation is indicated when the estimated PSE is smaller than the standard duration. Similarly, temporal underestimation is indicated when the PSE is larger compared to the standard duration. In this experiment, we used 550 ms as the standard duration. Therefore, we felt that it would be interesting to test whether the numerical magnitude genuinely affected temporal perception. In other words, we set out to test whether the estimated PSE for each numerical magnitude was significantly different from the standard duration. One can assume that if the numerical magnitude directly interacts with temporal processing, the numerical magnitude would cause a significant deviation in duration perception from the standard duration itself. To test this, we did a one-sample *t*-test and compared the estimated PSE for each magnitude against the standard duration, i.e., 550 ms, taken as the target value. The results of the one-sample *t*-test suggested that the PSE for Small (554.88 ± 79.8 ms), Identical (582.66 ± 84.8 ms), and large (523.54 ± 65.8 ms) magnitudes did not differ significantly from the standard duration, i.e., 550 ms (*p* > 0.05) suggesting that the numerical magnitude affected temporal perception in relative terms but may not have altered temporal processing itself concerning the objective duration.

### Does Numerical Magnitude Affect Duration Discrimination?

To test whether numerical magnitude affected temporal sensitivity, we calculated the Weber ratio for each numerical magnitude. Weber ratio is an index of temporal sensitivity, i.e., the Difference Limen ((*D*(*p*(long)) = 0.75 *D*(*p*(long)) = 0.25)/2) divided by standard duration. The lower the Weber ratio, the steeper the curve, and the higher the temporal sensitivity. The calculated weber ratio was analyzed using Friedman ANOVA. The results indicate that the temporal sensitivity did not differ across the three numerical magnitudes ($\chi_{\left(2\right)}^{2}$ = 2.33, *p* > 0.05), indicating that the numerical magnitudes did not help in discriminating the duration to be longer or shorter instead they might have biased the temporal perception. Further, to examine the null result of temporal precision we used *Bayesian RM ANOVA* using *JASP 0.12.2* to test whether the Weber ratio across three numerical magnitudes significantly differed from one other. The Bayes factor analysis yielded a value of *B*_10_ = 0.146, considering that it is below 1, we can conclude that there is favorable evidence for rejecting the alternative hypothesis (in other words, the results are 6.85 times more likely to have occurred under the null model).

## Discussion

In the present study, we investigated the influence of task-irrelevant numerical magnitudes on temporal perception using a *temporal discrimination task*. We proposed that if number and time are processed through a common magnitude system, we would observe differences both in temporal accuracy and temporal precision. Our experimental data indicate that while the numerical magnitude might bias our temporal judgments, it did not change the precision itself. The additional analysis supports that numerical magnitude may not directly affect temporal processing but could influence *via* attentional mechanisms.

### Do Time and Number Require Common Magnitude Processing?

**Figure 3:**
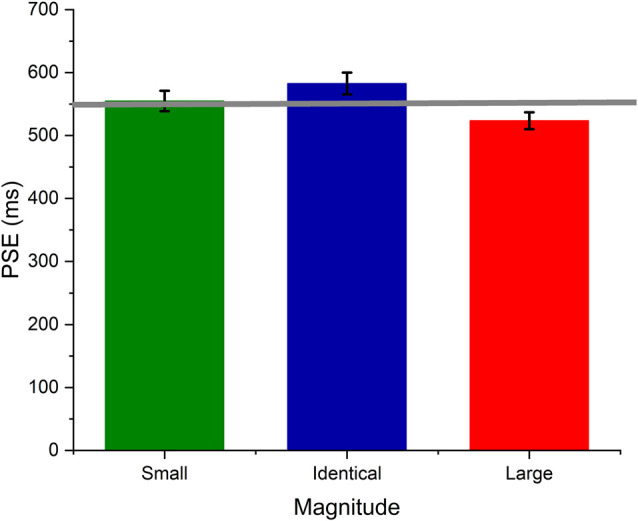
Average PSE values of small (1), Identical (5), and large (9) numerical magnitude trials. The error bar represents the standard error of the mean.

Several studies support the notion of the common magnitude system and extend the idea across various magnitude dimensions (Xuan et al., 2007; Srinivasan and Carey, 2010; Cai and Connell, 2015; Schwiedrzik et al., 2016; Yamamoto et al., 2016). On the contrary, many studies found substantial evidence against the existence of and need for a generalized magnitude system (Agrillo et al., 2010; Young and Cordes, 2013; Hamamouche et al., 2018). Our experimental data replicated the classical number effect on temporal processing, suggesting that duration is overestimated for the trials containing a large numerical magnitude than those containing small and identical numerical magnitudes. At the first glance, these results seem to support ATOM’s main predictions. However, when we analyzed our data beyond the relative magnitude effect, the PSE for each magnitude did not differ significantly from the standard duration (see Figure 3). This raises an interesting question as to whether indeed the numerical magnitude affects temporal processing genuinely, or the observed difference across different numerical magnitudes might always be in relative terms and may have occurred from the differential engagement of the cognitive processes required in processing the task-irrelevant magnitudes. Our experimental data indicate that influence of numerical magnitude on temporal processing is purely relative and may not require positing a generalized magnitude system. If the common magnitude system processes numerical magnitude and time, then we should have observed differences in PSE values not just in the relative sense but also when compared against the standard duration. This indicates that numerical magnitude may not change the temporal experience but perhaps biases temporal judgments in the presence of relative magnitudes.

### Can the Influence of Numerical Magnitude on Time be Explained by a Clock Mechanism?

Previous studies using the temporal reproduction paradigm have suggested that participants reproduced longer duration for large numerical magnitudes and shorter duration for the small numerical magnitudes. It has also been argued that numerical magnitude may affect the speed of the *internal clock*. Thus, large numerical magnitude causes speeding-up of the internal clock, and small numerical magnitude may slow down the speed of the internal clock. Consequently, the speeding-up or slowing-down of the internal clock might have affected the reproduction duration significantly. In contrast, our proportion of long response [*p(long)*] results do not support the idea of the acceleration of clock speed but suggest that numerical magnitude did not modulate temporal experience (partially supported by the analysis of PSE values as well) across durations.

**Figure 4:**
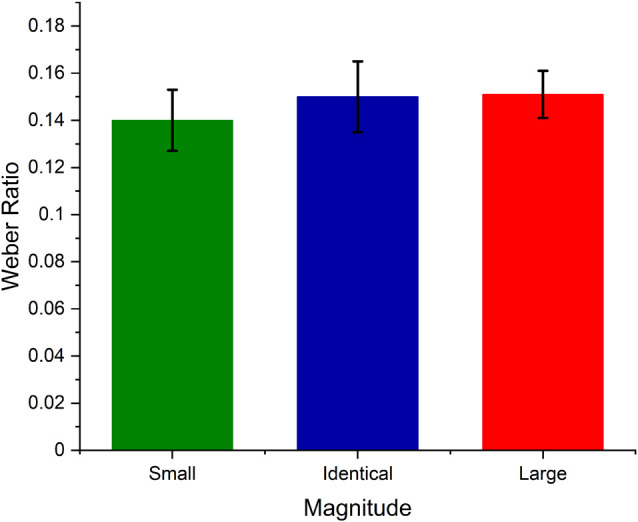
Average Weber ratio values of small (1), Identical (5), and large (9) numerical magnitude trials. The error bar represents the standard error of the mean.

Further, the Weber ratio analysis also provides indirect evidence against the common magnitude system. It has been argued that space, time, and a number have a cross-domain monotonic relation and therefore these magnitude dimensions can be mapped onto each other. If this is the case, then such cross-domain monotonic relation should affect the temporal discriminability resulting in differential temporal sensitivity across the three numerical magnitudes. However, our results suggest no temporal sensitivity differences across small, identical, and large numerical magnitudes (see Figure 4). This again suggests that number-time magnitude interaction may not arise from a change in temporal precision but could be the result of the change in temporal accuracy.

### If Not, Then why Does Numerical Magnitude Change Temporal Perception?

The results of the present study indicate that numerical magnitude and time may not need a common magnitude processing system. Partly, we have replicated the effect in a broader perspective, and when looked at other measures of temporal processing, numerical magnitude did not seem to influence the temporal experience. On the contrary, numerical magnitudes could bias temporal perception while making temporal judgments in more relative terms. Such relative temporal perception may be attributed to the automatic processing of numbers requiring differential attentional mechanisms that get engaged with differing numerical magnitudes. There seems to be some evidence to this from the results of previous research studies where small and large numerical magnitudes were either presented in a blocked or intermixed condition (Vicario, 2011). It has been observed that numerical magnitude affected temporal processing only when the numbers were presented in an intermixed order but not when presented in a separate block. Such effects have been attributed to the differential attentional requirements for the processing of the relative numerical magnitude. Thus, we suggest that the differential temporal perception observed in our experiments could also be due to the modulation of general attentional mechanisms involved in the automatic processing of numerical magnitude dimension (or numbers).

Specifically, spatial attention could play a role in mediating number and time interaction through the mental number/timeline. For example, large magnitudes (number and time) are associated with the right side of space, and small magnitudes (number and time) to the left side of space. Evidence from the number processing studies shows that the mere presence of numbers (small or large) induces a shift of attention (leftward or rightward, respectively) in the mental space (Fischer et al., 2003). Similarly, temporal durations are affected by direct manipulation of visuospatial attention using optokinetic stimulation. The leftward optokinetic stimulation resulted in temporal contraction. In contrast, rightward optokinetic stimulation causes temporal expansion (Vicario et al., 2007). Similarly, in another study, when number and time magnitude were presented in the left and right visual space, authors noted that independent of the numerical magnitude, temporal underestimation was observed when stimuli were on the left and overestimation when stimuli were on the right visual space. However, temporal estimation was biased by a numerical magnitude when the numbers were presented at the center of the visual space (Vicario et al., 2008). Thus, these results suggest a role for spatial attention in processing numerical and temporal information.

## Conclusion

The present study investigated whether number-time interaction arises from the change in temporal accuracy or temporal precision or both. Our data suggest that the temporal accuracy (judgment) is biased by the presence of numerical magnitude but did not modulate temporal precision (discrimination) itself. We suggest that such biases can occur from the attentional mechanism and may not be contingent on the existence of a common magnitude processing system proposed under the ATOM framework.

## Data Availability Statement

The raw data supporting the conclusions of this article will be made available by the authors, without undue reservation.

## Ethics Statement

The studies involving human participants were reviewed and approved by Institute Review Board (IRB), International Institute of Information Technology, Hyderabad, India. The patients/participants provided their written informed consent to participate in this study.

## Author Contributions

AS conceptualized and designed the experimental paradigm, collected raw data, as well as analyzed and interpreted the results. RSB interpreted the results. AS and RSB wrote the manuscript. All authors contributed to the article and approved the submitted version.

## Conflict of Interest

The authors declare that the research was conducted in the absence of any commercial or financial relationships that could be construed as a potential conflict of interest.
